# COVID-19 causing acute deterioration of interstitial lung disease: a case report

**DOI:** 10.1186/s43055-021-00431-2

**Published:** 2021-02-08

**Authors:** Mohamed Rafi Kathar Hussain, N. Kulasekaran, A. M. Anand, Padma Reka Danassegarane

**Affiliations:** Department of Radio Diagnosis, Sri Manukula Vinayagar Medical College, Puducherry, India

**Keywords:** COVID-19, Interstitial lung disease, Computerized tomography, Acute deterioration

## Abstract

**Background:**

Interstitial lung disease (ILD) comprises a heterogeneous group of disorders characterized by multifocal diffuse lung involvement. Similarly, COVID-19 has varied multispectral organ involvement. Patients with underlying ILD and coexistent COVID-19 infection may lead to an acute blow to the already deceased lung.

**Case presentation:**

A 58-year-old man presented with fever and cough with expectoration for the past 4 days associated with breathlessness. He was a smoker and alcoholic for the past 20 years. His saturation was low in room air around 84% and improved to 98% with 10 l/min of nasal oxygen. Further investigation shows acute deterioration of ILD.

**Conclusion:**

COVID-19 infection has a spectrum of manifestations. Acute deterioration of ILD is rarely reported in the literature. Etiology should be investigated further.

## Background

Interstitial lung disease (ILD) comprises a complex group of disorders characterized by multifocal diffuse lung involvement. Likewise, COVID-19 infection had a different pattern involvement with a multi-spectrum of disease exemplar. Patients with ILD after COVID-19 infection may lead to an acute blow to the already deceased lung. COVID-19 is a global pandemic caused by severe acute respiratory syndrome coronavirus 2 (SARS-CoV2). Post infective pulmonary fibrosis is common after any viral pneumonia [1]. The most common pulmonary imaging manifestation of the coronavirus is ground-glass opacity (GGO) and consolidation which is usually bilateral, sup-pleural, and peripheral [2, 3]. The clinical presentation of the coronavirus ranges from mild asymptomatic to severe infection. The majority of mild to moderate illness cases are completely recovered whereas a small proportion of severely infected cases remain in hypoxemia, despite adequate medical management [4]. COVID-19 infects all people irrespective of age, gender, race, and those with or without co-morbidities like ILD.

## Case presentation

A 58-year-old man presented with complaints of fever and cough with expectoration for the past 4 days associated with breathlessness. He was a known smoker and alcoholic for the past 20 years. He is not a known diabetic or hypertensive. The patient was diagnosed to be RT-PCR positive for coronavirus 45 days before and treated in an outside hospital for COVID-19 infection. Repeat RT-PCR in our hospital, at the time of admission, was found to be negative. He was a known case of ILD of combined pulmonary fibrosis and emphysema (CPFE) type on irregular treatment. He was admitted to an outside hospital for treatment of COVID-19 infection and discharged. Data regarding this outside hospital stay and treatment undertaken was not available. CT imaging done during this stay could not be retrieved.

On examination, there was no pallor, icterus, cyanosis, clubbing, or lymphadenopathy. His blood pressure was 130/90 mm Hg, and his respiratory rate was 26 breaths/min. Heart rate was 130 bpm. His saturation was low in room air around 84% and improved to 98% with 10 l/min of nasal oxygen. Lab parameters reveal normal random blood glucose. Serum urea and blood creatinine were within normal limits. The total count was reduced to 3000 indicating leukocytopenia. His eosinophil counts were within normal limits.

**Fig. 1:**
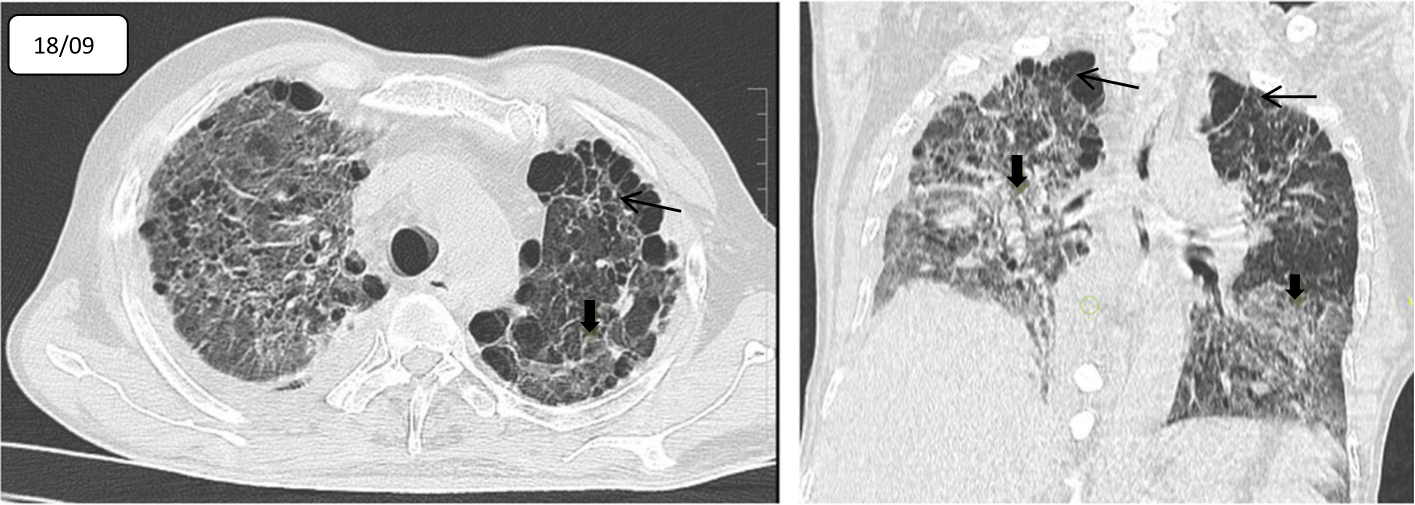
**a**, **b** An initial HRCT axial and coronal section shows diffuse ground glass opacities and reticular interstitial thickening (solid black arrow) with emphysematous changes and bulla (line arrow) in bilateral lung fields

On admission, HRCT thorax was done and showed diffuse interstitial thickening and ground-glass opacities in bilateral lung parenchyma associated with diffuse emphysematous changes and bullae predominantly in the bilateral upper lobes, consistent with interstitial lung disease (Fig. 1). The patient was started on oral methylprednisolone. Despite adequate medical management, the patient was persistently dependent on oxygen supplementation with continuing symptoms. His stool examination revealed *Strongyloides* infection, and hence he was administered with two doses of 12 mg ivermectin. Pulmonary *Strongyloides* was not suspected because the blood eosinophil count was within normal limits. Treatment continued along with the oxygen supplementation for ILD.

**Fig. 2:**
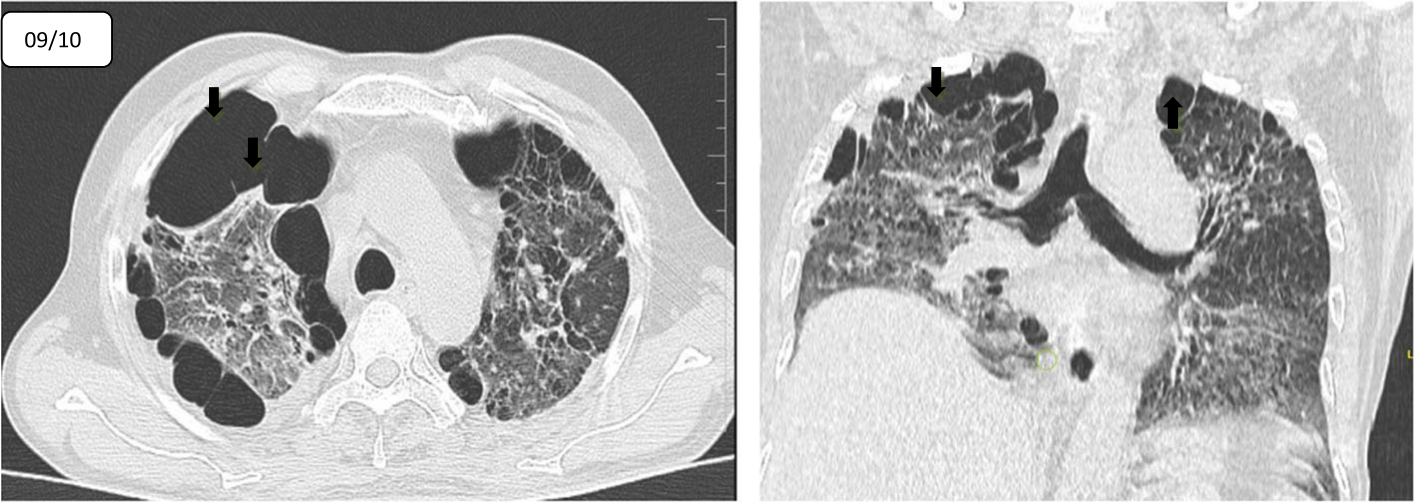
**a**, **b** Repeat HRCT after 1 month axial and coronal sections shows marked increase in emphysematous and bullous changes (black arrow) with reticular thickening and ground glass opacities

Repeat CT thorax after a month showed a marked increase in emphysematous and bullous changes in the bilateral upper lobes when compared to the previous CT (Fig. 2). There were also bilateral subpleural fibrotic bands are seen in the basal segments of the bilateral lower lobes. Because of post-COVID-19 pulmonary fibrosis, he was started on pirfenidone. The patient showed mild improvement over the next few days and was discharged home requiring 4 l/min of oxygen supplementation. And the patient was asked to have strict follow-up and close monitoring.

**Fig. 3:**
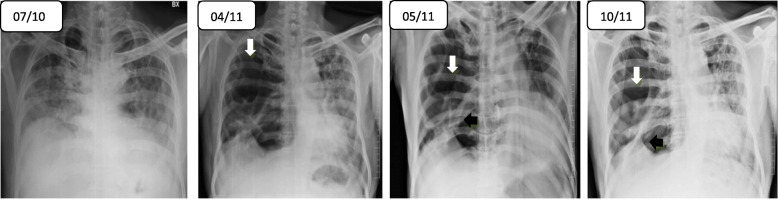
**a**–**d** Consecutive chest x-ray PA view shows progressive increase in lucencies in the right hemithorax (white arrow) with fibrotic changes (black arrow) and mediastinal shift towards the left

One week after discharge, the patient was again re-hospitalized due to worsening dyspnea and increased cough. Chest radiographs was done and showed serial significant progression of emphysematous changes (Fig. 3). The patient was again started on methylprednisolone for flaring up of interstitial lung disease. Despite starting on steroids and with 10 l/min of nasal oxygen, his condition worsened and ultimately requiring non-invasive ventilation and hence transferred to medical ICU for vigorous monitoring. Although many factors like smoking and alcohol can also cause acute deterioration of ILD, in the present clinical contest of recent COVID-19 infection, all other factors are excluded. He has been diagnosed as a case of a post-COVID-19 acute deterioration of ILD. At the time of writing this article, the patient was in ICU under oxygen support.

## Discussion

The acute stage of lung infection in COVID-19 presents with ground glass opacities whereas the late-stage manifest as crazy pavy patterns and subpleural peripheral fibrotic bands. Some COVID-19 infection leading to progressive fibrotic lung disease was also reported in the literature. Anti-fibrotic drugs like pirfenidone and nintedanib in some cases had been proven to be beneficial [5]. The pathogenesis of post-viral pulmonary fibrosis is most likely due to the release of matrix metalloproteinase causing epithelial and endothelial injury [6]. Vascular endothelial growth factor, interleukin-6, and TNF- alpha are also being implicated in pulmonary fibrosis [7]. There are many etiologies for lung fibrosis; one of the causes is interstitial lung disease (ILD).

ILD is a heterogeneous group of disorders that are classified into four types: (a) ILD with a known cause like rheumatoid arthritis, asbestosis, connective tissue disorders, and hypersensitive pneumonitis; (b) idiopathic interstitial pneumonia (IIP) like idiopathic pulmonary fibrosis, nonspecific interstitial pneumonia (NSIP), respiratory bronchiolitis associated ILD (RB-ILD), desquamative interstitial pneumonia (DIP), and acute interstitial pneumonia; (c) granulomatous ILD—sarcoidosis and silicosis; and (d) others include Langerhans cell histiocytosis (LCH) and lymphangioleiomyomatosis [8]. The common patterns of findings in ILD include a combination of reticulation, ground-glass opacities, honeycombing, traction bronchiectasis, nodules, and thickening of interlobular septa [9].

The most common idiopathic interstitial pneumonia includes usual interstitial pneumonia (UIP) and NSIP. UIP has typical findings of reticulation, bilateral subpleural symmetrical basal distribution of honeycombing [10]. The key finding in NSIP includes bilateral symmetrical ground-glass opacities in lower lobe predominance with subpleural sparing. Overtime, GGO can be replaced by reticulation [11]. The cardinal features of RB-ILD include centrilobular nodules, GGO, and smooth thickening of interlobular septa, distributed in the upper lobes. The imaging features of desquamative interstitial pneumonia are GGO in the mid and lower zones [8]. Smoking-related ILD includes RB-ILD, LCH, DIP, and combined pulmonary fibrosis with emphysema (CPFE). CPFE is characterized by the co-existence of UIP/NSIP with emphysema in smokers. The emphysematous changes in CPFE are often predominant in the upper zone.

The co-existence of interstitial lung disease and COVID-19 has been reported, and it has been postulated that patients with COVID-19 have an increased risk for developing interstitial lung disease [12]. In patients with preexisting ILD, COVID-19 infection has led to acute exacerbation of underlying ILD. The criteria for ILD exacerbation include subacute worsening of dyspnea and hypoxemia, new pulmonary infiltrates on imaging, and absence of pulmonary emboli, cardiac failure, and other non-pulmonary causes. Surgeries, aspiration of gastric contents, infection, and other factors have also been postulated for ILD exacerbation [13].

ILD renders the host susceptible to viral infection, although the exact etiology is unknown [14]. ILD exacerbation has the worst prognosis [15]. Patients with ILD exacerbation are treated with corticosteroids/other immunosuppressants. It was presumed that if the steroid is given to patients with COVID-19, it decreases the resistance against the virus [13]. To date, there are no clear data to support this fact. A recent study postulated that steroids were likely to be safe and beneficial in patients with severe respiratory disease [16]. In a case-control study by Esposito et al., patients with ILD in advanced age has increased the odds of the worse outcome in COVID-19 patients with underlying ILD [17].

## Conclusion

In this current pandemic scenario, COVID-19 is causing major mortality and morbidity in the entire world. COVID-19 infection in the preexisting ILD patient causes acute aggravation of lung changes in imaging and clinical deterioration. And this needed further research. ILD is the chronic lung condition of the variable progressive poor prognosis. COVID-19 infection on ILD patients is like whipping the already tired horse which will unquestionably lead to increased mortality and morbidity.

## Data Availability

Available.

## Abbreviations

ILD: Interstitial lung disease
HRCT: High-resolution computed tomography
SARS-CoV2: Severe acute respiratory syndrome coronavirus 2
IIP: Idiopathic interstitial pneumonia
RB-ILD: Respiratory bronchiolitis-associated ILD
GGO: Ground glass opacities
DIP: Desquamative interstitial pneumonia
CPFE: Combined pulmonary fibrosis with emphysema
UIP: Usual interstitial pneumonia
NSIP: Nonspecific interstitial pneumonia
LCH: Langerhans cell histiocytosis
